# Polysaccharides Derived From the Brown Algae *Lessonia nigrescens* Enhance Salt Stress Tolerance to Wheat Seedlings by Enhancing the Antioxidant System and Modulating Intracellular Ion Concentration

**DOI:** 10.3389/fpls.2019.00048

**Published:** 2019-01-31

**Authors:** Ping Zou, Xueli Lu, Hongtao Zhao, Yuan Yuan, Lei Meng, Chengsheng Zhang, Yiqiang Li

**Affiliations:** ^1^Marine Agriculture Research Center, Tobacco Research Institute of Chinese Academy of Agricultural Sciences, Qingdao, China; ^2^State Key Laboratory of Bioactive Seaweed Substances, Qingdao, China

**Keywords:** salt tolerance, seaweed polysaccharide, *Lessonia nigrescens*, molecular weight, sulfate content

## Abstract

Soil salinity reduces plant growth and is a major factor that causes decreased agricultural productivity worldwide. Seaweed polysaccharides promote crop growth and improve plant resistance to abiotic stress. In this study, polysaccharides from brown seaweed *Lessonia nigrescens* polysaccharides (LNP) were extracted and further separated and fractionated. Two acidic polysaccharides (LNP-1 and LNP-2) from crude LNP were obtained and characterized. The latter had a lower molecular weight (MW) (40.2 kDa) than the former (63.9 kDa), but had higher uronic acid and sulfate content. Crude LNP and LNP-2 were composed of mannose, glucuronic acid, fucose, and xylose, whereas LNP-1 has little mannose. Moreover, the effects of the three polysaccharides on plant salt tolerance were investigated. The results showed that crude LNP, LNP-1, and LNP-2 promoted the growth of plants, decreased membrane lipid peroxidation, increased the chlorophyll content, improved antioxidant activities, and coordinated the efflux and compartmentation of intracellular ion. All three polysaccharides could induce plant resistance to salt stress, but LNP-2 was more effective than the other two. The present study allowed to conclude that both MW and sulfate degree contribute to salt resistance capability of polysaccharides derived from *L. nigrescens*.

## Introduction

Soil salinization is a major environmental problem that restricts crop growth and yield worldwide, mainly due to osmotic stress and ion toxicity (Rivero et al., 2014). Salinization is predicted to degrade approximately 30% of all cultivated land by 2050 (Van Oosten et al., 2017). High concentrations of salts in the soil decrease the capacity of roots to extract water, and high concentrations of salts in the plant can be toxic (Munns and Tester, 2008; Annunziata et al., 2017). High salt concentration in the soil has a devastating effect on plant metabolism, disrupting cellular homeostasis and uncoupling major physiological and biochemical processes. Salinity stress also results in production of reactive oxygen species (ROS) in plants causing oxidative stress. Along evolution, plants have evolved a ubiquitous mechanism of salinity resistance, involving synthesis and accumulation of compatible compounds, sodium sequestration in vacuoles, and enhancing antioxidant enzymes activities. These specific strategies regulate cell penetration and control ion and water homeostasis to minimize stress damage and sustain growth.

Salinity resistance mechanisms can be further improved through the application of exogenous biostimulants (Bose et al., 2014). A plant biostimulant is a substance or microorganism that is beneficial to plants and can enhance nutrient uptake and nutrient use efficiency, abiotic stress tolerance, and crop quality, regardless of its own nutrient content. Considering that crops are exposed to extreme temperatures, drought and soil salinization, biostimulants may contribute more and more to global crop yields and quality as growth promoters and stress protectors. Various biostimulants have been used in commercial agriculture, such as humic and fulvic acids, protein hydrolysates and other N-containing compounds, seaweed extracts and botanicals, chitosan and other biopolymers, inorganic compounds, beneficial fungi, and bacteria (Jardin, 2015). Seaweed extracts have long been used in agriculture as sources of organic matter and as fertilizer. The beneficial constituents contributing to the plant growth promotion include the polysaccharides, micro- and macronutrients, sterols, N-containing compounds like betaines, and hormones. Many studies have demonstrated that polysaccharide from seaweed is a kind of plant physiological stimulants and stress-resistance elicitors. In tobacco plants, for example, carrageenan application can activate defense systems involving ethylene, jasmonic acid, and salicylic acid pathways in tobacco plants, while weekly spraying of 1 mg/ml oligocarrageenans stimulated growth, photosynthesis, and basal metabolism (Castro et al., 2011). The bioactive components in *Ulva lactuca* (Ibrahim et al., 2014) and *Fucus spiralis* (Latique et al., 2017) extract could enhance in the percentage of seed germination and growth parameters. Chernane et al. (2015)’s study suggested that seaweed extract of *Ulva rigida* can improve salt stress tolerance and contribute to protection of wheat plant against oxidative deterioration. Currently, the primary algal polysaccharides on the phytosanitary market are laminarans, derived from brown algae [e.g., *Laminaria digitata* (Hudson) J.V.]. Laminarans can induce various defense responses in tobacco and grapevine cell suspensions, including protein kinase activation, Ca^2+^ influx, oxidative outburst, extracellular-media alkalinization, and phytoalexin production. When sprayed on tobacco and grapevine plants, laminarans stimulate phytoalexin accumulation and expression of PR-proteins (Klarzynski et al., 2000; Aziz et al., 2003). The ability of these algal polysaccharides to activate multiple plant defenses is likely to benefit the development of novel resistance inducers. Economically important algae can be found in rocky intertidal and shallow subtidal zones, contain numerous bioactive compounds (e.g., fucans and phlorotannins) (Gonzalez et al., 2012). One of these species, *Lessonia nigrescens* Bory de Saint-Vincent grows quickly and produces large biomass, indicating its potential for agricultural application. However, the effectiveness of *L. nigrescens* compounds for stimulating the resistance of cultivated plants remains unclear. The purpose of the present study was to assess the effects of *L. nigrescens* polysaccharides (LNP) on wheat seedlings under salt stress. Moreover, we aimed to contribute to the understanding of the regulatory mechanism of LNPs in the improvement of plant salt stress resistance in terms of osmotic regulation, ion transport, and redox homeostasis. This study provides a simple, efficacious, and sustainable approach to ameliorate salt stress in commercially important crops.

## Materials and Methods

### Samples and Reagents

Dried *L. nigrescens* was supplied by State Key Laboratory of Bioactive Seaweed Substances (Qingdao, China). After being ground, the seaweed was sieved through a 0.45 mm sifter and stored in a desiccator. Standard sugars were purchased from Sigma (United States). All other chemicals and reagents were of analytical grade.

### Extraction of Crude Polysaccharides

*Lessonia nigrescens* (100 g) was extracted with 80% ethanol (2 l) under mechanical stirring at room temperature for 24 h to remove pigments, proteins, salts, and other small molecules. Next, 50 g of the dried residue was extracted with 1.5 l 0.1 M HCl in a 3 l flask at 100°C for 2 h. The precipitate was removed using gauze, and the remaining supernatant was filtered using siliceous earth. Subsequently, 2% (w/v) CaCl_2_ solution was added to the liquid fraction, and the mixture was maintained overnight at 4°C for alginate removal and was then separated by centrifugation. The filtrate was dialyzed against distilled water for 48 h and concentrated under reduced pressure to approximately one-fourth of its original volume. Finally, polysaccharides were precipitated using fourfold volume of ethanol and were then lyophilized to yield LNP.

### Purification of LNP Fraction

Crude polysaccharide (10 mg) solution (10 mg/ml) was loaded onto a DEAE-52 anion exchange column (2.6 × 30 cm). The column was eluted with a stepwise gradient of distilled water, followed by 0.1, 0.2, 0.3, 0.4, and 0.5 M NaCl solution at a flow rate of 1.0 ml/min. The eluate (10 ml/tube) was collected automatically (BSZ-100, Shanghai QingpuHuxi Instrument Factory Co., Ltd., P.R. China). Polysaccharide fractions were analyzed using the phenol–sulfuric acid method, eventually yielding two fractions.

These fractions were then re-dissolved in distilled water and loaded onto a Sephadex G-100 gel column (1.6 cm × 100 cm) for a second elution (0.1 M NaCl at a flow rate of 20 ml/h). As before, the eluent (5 ml/tube) was collected automatically and analyzed. The two purified fractions (LNP-1 and LNP-2) were concentrated, dialyzed, and lyophilized for further analyses.

### Chemical Composition

#### Determination of Carbohydrate, Protein, Sulfate, and Uronic Acid Content

Total carbohydrate content of polysaccharides was determined via the phenol–sulfuric acid method, using D-glucose as a standard (Chen C.F. et al., 2018). Protein content was determined by the method of Bradford, with bovine serum albumin as a standard (Chen G. et al., 2018). The sulfate content was quantified using the BaCl_2_ gelation method (standard: K_2_SO_4_) (Palanisamy et al., 2018). Finally, uronic acid content was determined using the carbazole method, with glucuronic acid as a standard (Palanisamy et al., 2018).

#### Determination of Monosaccharide Composition

Polysaccharides (1 mg) were hydrolyzed in 0.5 ml of 2 M trifluoroacetic acid (TFA) at 120°C for 2 h. The resulting solution was dried on a nitrogen blowing apparatus. After TFA was removed, the hydrolysate was derivatized with 1-phenyl-3-methyl-5-pyrazolone (PMP) at 70°C for 30 min. The solution was analyzed using an Agilent 1260 Infinity HPLC instrument with a Thermo ODS-2 C18 (4.6 × 250 mm, 5 μm) column at 25°C, with a flow rate of 1 ml/min. Separated monosaccharides were quantified through external calibration with an equimolar mixture of nine monosaccharide standards (mannose, rhamnose, fucose, glucose, galactose, glucuronic acid, galacturonic acid, arabinose, and xylose) (Yuan and Macquarrie, 2015).

#### Determination of Average Molecular Weight

The average polysaccharide molecular weight (MW) was determined using an Agilent 1260 gel permeation chromatograph (Agilent Technologies, United States) fitted with a refractive index detector (Zou et al., 2018). Chromatography was run on a TSK G4000-PW_xl_ column with 0.05 M aqueous NaNO_3_ as the mobile phase. The flow rate was 0.5 ml/min and the column temperature was 30°C. Standards used to calibrate the column were dextrans with MWs of 1000, 5000, 12,000, 25,000, 50,000, 80,000, 270,000, and 670,000 Da (Sigma, United States).

#### Infrared Spectroscopy of Polysaccharides

Fourier transformed infrared (FT-IR) spectra of polysaccharides were plotted using a Thermo Fisher Scientific Nicolet iS10 FT-IR spectrometer (Thermo Fisher Scientific, United States) with KBr disks.

### Determination of Salt-Defense Elicitor Activity

#### Plant Material and Treatments

Wheat (*Triticum aestivum* L. Jimai 22) seeds were surface-sterilized with a 1% (v/v) sodium hypochlorite solution for 10 min and thoroughly rinsed with distilled water. Seeds were germinated for 24 h at 25°C and then sown on nylon mesh in Petri dishes containing Hoagland solution. Dishes were placed in a growth incubator under the following controlled conditions: 14 h light /10 h dark photoperiod, 25°C/20°C day/night cycle, 65% relative humidity, and photosynthetic photon flux intensity of 800 mmol m^-2^ s^-1^. Wheat seedlings with fully expanded second leaves were randomly divided into five groups. The experiment included five treatments: control (neither LNPs nor NaCl), negative control (150 mM NaCl), and three different LNP–NaCl mixtures. Each group contained three petri dishes, each with 30 plants. Nutrient solutions were renewed daily.

#### Growth Parameters

After 10 days of salt stress, three samples were randomly selected from each group and their physiological indices were determined. Next, 30 plants were randomly chosen and harvested to measure shoot length, root length, and fresh weight (FW). Samples were dried at 105°C for 2 h to determine dry weight (DW).

#### Determination of Lipid Peroxidation and Electrolyte Leakage

Membrane permeability was assessed by measuring relative electric leakage (REL) (Li et al., 2017). Fully expanded second leaves (1.0 g) were cut into 0.5-cm pieces and placed in a 50-ml test tube containing 30 ml distilled water. Leaf samples were then vacuumed for 30 min, immersed and vibrated for 20 min, before solution conductivity (EC1) was measured (DDSJ-308A, Shanghai Instrument and Electrical Scientific Instrument Ltd., Shanghai, China). Samples were then boiled for 30 min and cooled to room temperature. After this, conductivity (EC2) was measured again to yield REL (EC1/EC2 × 100%).

Malondialdehyde (MDA) content in plants indicates lipid peroxidation levels, and thiobarbituric acid (TBA) reactions were used to determine MDA content according to the previous method with certain modifications (Buono et al., 2011). 0.5 g leaf samples were homogenized in 10% (w/v) TCA before centrifugation at 4000 ×*g* for 10 min. Two milliliters of 0.6% (w/v) TBA was added to 2 ml of the supernatant, and the mixture was then heated in boiling water for 15 min cooled, then centrifuged at 10,000 ×*g* for 15 min. Absorbance (optical densities) were read at 450, 532, and 600 nm. MDA content was recorded as mg MDA/g FW.

#### Chlorophyll Contents

After 10 days of NaCl treatment, chlorophyll a (Chl a), chlorophyll b (Chl b), and total chlorophyll (Chla+b) content in seedlings were measured spectrophotometrically (665 and 649 nm), following previously published procedures (Ma et al., 2017). The procedure was performed under low light to avoid chlorophyll degradation.

#### Soluble Sugar Content and Proline Content

Leaf samples (0.5 g) were chopped and heated at 100°C in 5 ml distilled water for 30 min. Extracts were diluted fivefold. Extracts (500 ml), 5% (v/v) phenol (1 ml), and sulfuric acid (5 ml) were mixed, then left to stand for 5 min before absorbance was read at 485 nm (Zou et al., 2018). Soluble sugar concentration was quantified through comparisons against a glucose standard curve.

To determine proline content, 0.2 g leaf samples were ground in liquid nitrogen and homogenized in 5 ml of 3% (w/v) sulfosalicylic acid (Misra and Saxena, 2009). The sample was centrifuged at 18,000 ×*g* for 10 min 500 μl of filtrate and made up 1 ml with distilled water and thereafter 1 ml glacial acetic acid and 1 ml ninhydrin reagent added and then heated at 100°C for 10 min, cooled to room temperature, and centrifuged at 5000 ×*g* for 4 min. Proline content in the supernatant was determined spectrophotometrically at 520 nm.

#### Antioxidant Enzyme Activities

After salt stress for 10 days, fully expanded second leaves (0.5 g) were used to extract enzymes. Samples were homogenized in liquid nitrogen and brought up to a volume of 5 ml with 0.2 mol/l cold sodium phosphate buffer solution (pH 7.8). Homogenates were centrifuged at 12,000 ×*g* and 4°C for 15 min. Supernatants were immediately used to determine enzyme activities. The Bradford method was used to determine total soluble protein (Bradford and Williams, 1976). Superoxide dismutase (SOD) activity was assayed by the extent to which it inhibited the photochemical reduction of b-nitro blue tetrazolium chloride (NBT) (Zhang et al., 2016). Catalase (CAT) activity was determined based on the rate of disappearance of H_2_O_2_, as measured by decline in absorbance at 240 nm (Ma et al., 2017). Peroxidase activity (POD) was calculated from the rate of the formation of the guaiacol dehydrogenation product and was expressed as mmol GDHP min^-1^ mg^-1^ protein (Marta et al., 2016).

#### Measurement of Na^+^ and K^+^ Concentrations

Na^+^ and K^+^ concentration were measured as described previously (Zhang et al., 2018). Plant tissues, leaves, sheaths, and roots were dried at 60°C overnight. Dry samples (0.5 g) were incinerated in a muffle furnace at 500°C for 6 h. The ashes were dissolved in 5 ml of concentrated nitric acid with 500 ml distilled water. Ion concentrations were determined using an Atomic Absorption Spectrometer 900T (PerkinElmer, United States).

#### Expression Analysis of Genes Encoding Na^+^/K^+^ Transporter

Total RNA was extracted from leaves, sheaths, and roots of wheat seedlings using a Plant RNA Extraction Kit (Takara, Dalian, China). Total RNA was quantified using a UV spectrophotometer. First-strand cDNA was synthesized from 1 μg mRNA using a PrimeScript^TM^ RT Reagent Kit with gDNA Eraser (Takara, Dalian, China). Real-time quantitative PCR was performed using an ABI 7500 (Life Tech Applied Biosystems, United States) with a TB Green^TM^ Premix Ex TaqTM (Takara, Dalian, China). RT-PCR was performed in a total volume of 20 μl containing 100 ng of the first strand cDNA reaction products. Amplicons were subjected to melting curve analysis. Relative expression was analyzed using the comparative threshold cycle method (2^-ΔΔCt^), with β-actin as a housekeeping gene. Primers are listed in Supplementary Table S1.

### Statistical Analyses

All data are represented as means ± SD of three independent replicates. Analysis of variance (ANOVA) and *post hoc* Duncan’s tests (*P* < 0.05) were used to compare means across treatments.

## Results

### Purification of LNPs

**FIGURE 1:**
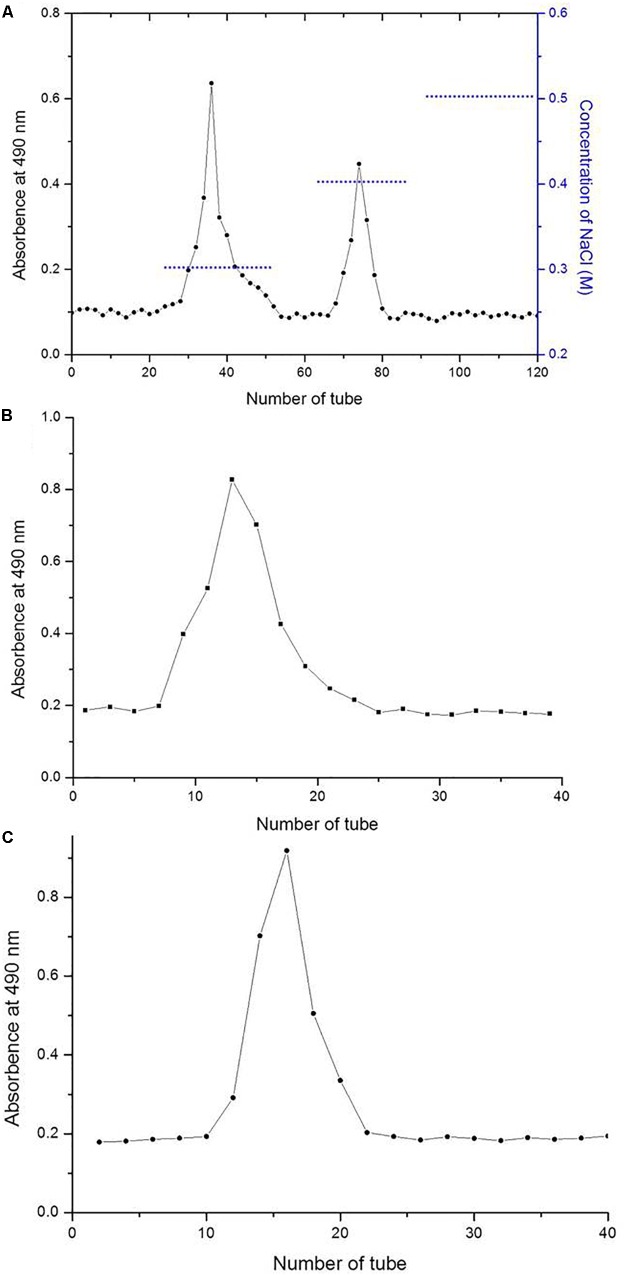
Elution profiles of *Lessonia nigrescens* polysaccharide (LNP) fractions on a DEAE-cellulose column **(A)** and Sephadex G-100 gel chromatography column (**B** and **C**).

The two LNP fractions (Figure 1A) detected during purification generated only one symmetrical peak, indicating relative homogeneity (Figure 1B,C). The two purified polysaccharides were termed LNP-1 (Figure 1B) and LNP-2 (Figure 1C). No fractions were obtained by elution with distilled water, which indicated the absence of neutral laminaran-type polysaccharides. This is consistent with the results of Nancy who found no laminaran-type polysaccharides in most species of the genus *Lessonia* (Nancy et al., 2001).

### Preliminary Characterization of LNPs

The MWs of LNP, LNP-1, and LNP-2 were 45.4, 63.9, and 40.2 kDa, respectively (Supplementary Figure S1). The three polysaccharides differed in the amount of carbohydrate, protein, uronic acid, and sulfate content (Table 1), with LNP showing a significantly lower carbohydrate content (67.4%) than either LNP-1 (87.3%) or LNP-2 (84.0%). This result indicates that impurities were removed from crude polysaccharides. Protein contents of the three polysaccharides were similarly low. Total uronic acid content was lower in LNP-1 (16.4%) and LNP-2 (20.1%) than in LNP (22.1%). In comparison, uronic acid content was slightly higher than that of the polysaccharides extracted with diluted HCl from *Lessonia* sp. (Phaeophyceae) (14.7%) (Leal et al., 2018). Sulfate content in LNP, LNP-1, and LNP-2 was 33.7, 36.9, and 40.5%, respectively. Similarly, extractions from *L. vadosa* with 2% aqueous CaCl_2_ produced fucoidan with 37.7% sulfate (Nancy et al., 2004).

**Table 1:** Preliminary characterization of crude LNP, LNP-1, and LNP-2.

Item	LNP	LNP-0.3	LNP-0.4
Molecular weight (kDa)	45.4	63.9	40.2
Carbohydrate (%)	67.4	87.3	84.0
Protein (%)	6.7	7.0	6.9
Uronic acid (%)	22.1	16.4	20.1
Sulfate	33.7	36.9	40.5
**Sugar components (%)**			
Mannose	0.24	0.05	0.29
Glucuronic acid	0.22	0.15	0.21
Galacturonic acid	0.02	0.06	0.03
Glucose	0.05	0.33	0.11
Galactose	0.13	0.09	0.06
Xylose	0.13	0.17	0.13
Fucose	0.21	0.15	0.17

The three polysaccharide fractions had different monosaccharide profiles (Table 1). Both LNP and LNP-2 contained mannose, glucuronic acid, fucose, xylose, galactose, and glucose. However, LNP contained more galactose, while LNP-2 contained more glucose. In contrast, LNP-1 was mainly composed of glucose, glucuronic acid, fucose, and xylose, with very little mannose. Notably, glucose content was highest in LNP-1. Previous studies on *Lessonia* sp. (Phaeophyceae) found that extracted polysaccharides were mainly fucose, with trace amounts of galactose and xylose (Leal et al., 2018).

**FIGURE 2:**
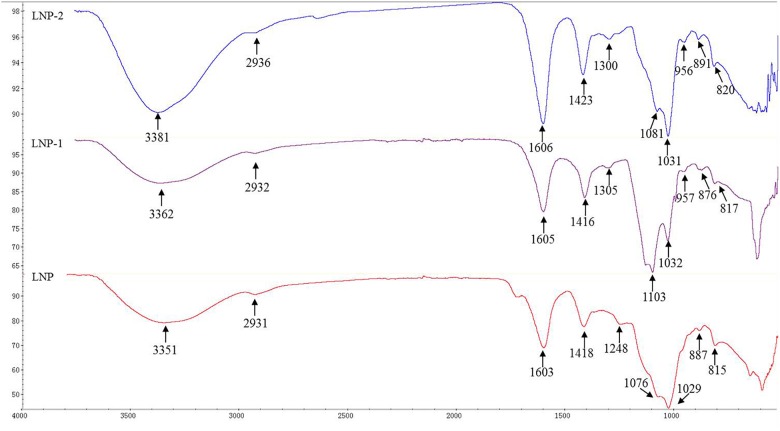
FT-IR spectra of LNP, LNP-1, and LNP-2.

Typical carbohydrate absorptions at 4000–500 cm^-1^ were observed in all three polysaccharides (Figure 2). Strong peaks around 3350–3380 cm^-1^ were assigned to hydroxyl stretching vibration. Weak peaks around 2930 cm^-1^ and 1420 cm^-1^ were characteristic of –CH stretching vibration and –CH_2_ bending vibration, respectively. Additionally, the strong extensive absorption around 1100–1000 cm^-1^ was due to C–O–C and C–OH stretching vibration. Bands around 1600 cm^-1^, corresponding to C–O double-bond asymmetric stretching vibration, were assigned to uronic acid carbonyl groups. A major peak at 1248–1300 cm^-1^ was attributed to the asymmetric stretching of S=O and suggested the presence of an ester sulfate (Yuan and Macquarrie, 2015). Previous studies have reported that sulfate groups at equatorial C-2 and C-3 positions produced bands around 820 cm^-1^ (Foley et al., 2011), and there is such a shoulder in the three polysaccharides at 815–820 cm^-1^. Absorption peaks at 876–891 cm^-1^ indicated the presence of primarily β-glycosidic linkages (Ye et al., 2016).

### Effect of Polysaccharides on Wheat Seedlings Under Salt Stress

#### Plant Growth and Biomass Accumulation

Under NaCl stress, the shoot length, root length, FW, and DW of wheat seedlings were all significantly lower than in control (Table 2). In contrast, all of these parameters increased due to treatment with LNP, LNP-1, and LNP-2.

**Table 2:** Effects of LNP, LNP-1, and LNP-2 on growth parameters of wheat seedlings.

	Shoot length (cm)	Root length (cm)	Fresh weight (g)	Dry weight (g)
control	28.6 ± 1.8^a^	19.8 ± 4.2^b^	0.89 ± 0.11^a^	0.096 ± 0.014^bc^
NaCl stress	20.8 ± 1.6d	17.0 ± 3.7c	0.74 ± 0.11c	0.088 ± 0.011c
LNP+NaCl stress	22.0 ± 1.5c	22.7 ± 3.4^a^	0.87 ± 0.11^a^	0.110 ± 0.012^a^
LNP-1+NaCl stress	21.8 ± 1.3c	21.9 ± 4.0ab	0.80 ± 0.09^b^	0.098 ± 0.018^b^
LNP-2+NaCl stress	23.3 ± 1.3^b^	23.3 ± 4.0^a^	0.87 ± 0.11^a^	0.115 ± 0.017^a^

*Values are mean ± SD of 30 replicates. Different letters indicate significant differences at *P* < 0.05.*

Compared to the NaCl stress treatment, exogenous application of LNP, LNP-1, and LNP-2 increased wheat seedling shoot lengths by 6.1, 4.7, and 12.0%, respectively. Treatment with LNP, LNP-1, and LNP-2 significantly increased wheat seedling root length by 33.5, 29.0, and 37.3%, respectively, compared to the negative control. No statistically significant differences of wheat seedling shoot length were observed among polysaccharide treatments. The three polysaccharide treatments also increased the fresh and DWs of wheat seedlings under NaCl stress. LNP, LNP-1, and LNP-2 increased wheat seedling FWs by 18.1, 8.1, and 17.7% and their DWs by 25.0, 11.8, and 30.9%, respectively, compared to the NaCl stress treatment. The fresh and dry seedling weight in the LNP and LNP-2 groups were significantly higher than those in the LNP-1 group. These results suggest that the application of any of the LNPs can improve wheat seedling growth parameters (Supplementary Figure S2). Of the three applied polysaccharides, LNP and LNP-2 were more effective than LNP-1 for improving the growth parameters of plants under salt stress.

#### Lipid Peroxidation

**FIGURE 3:**
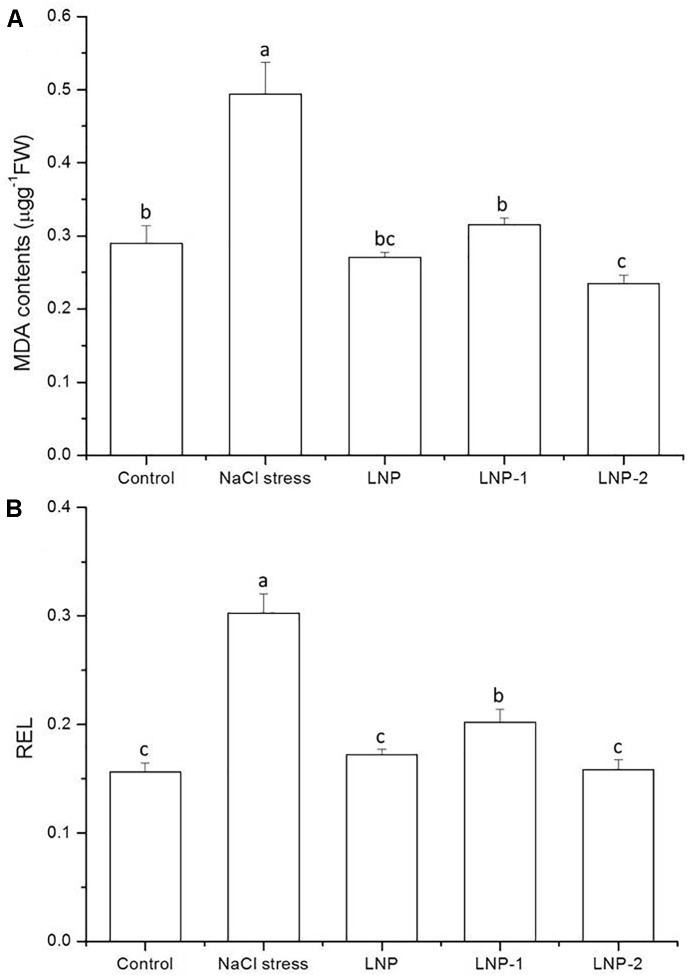
Effect of LNP, LNP-1, and LNP-2 on MDA content **(A)** and REL **(B)** in leaves of wheat seedlings. Values are the mean ± SD of three replicates. Different letters indicate significant differences at *P* < 0.05. MDA, malondialdehyde; REL, REL.

Increased soil salinity can cause cell membrane impairment in plants by elevating the amount of ROS. A significant increase was observed in MDA content in wheat seedling leaves under salt stress (Figure 3A), and this oxidative damage can be alleviated by exogenous application of LNPs. LNP, LNP-1, and LNP-2 reduced the MDA content to 45.1, 36.2, and 52.4% compared to NaCl-stressed plants. Similarly, the REL significantly increased by 94.2% due to NaCl stress (Figure 3B). LNP, LNP-1, and LNP-2 significantly reduced the REL to 43.1, 33.4, and 47.7% compared to the NaCl-treated group. Moreover, LNP-2 significantly reduced the MDA content and REL in the seedlings compared to NaCl-stressed plants more than the LNP-1.

#### Chlorophyll Content

**FIGURE 4:**
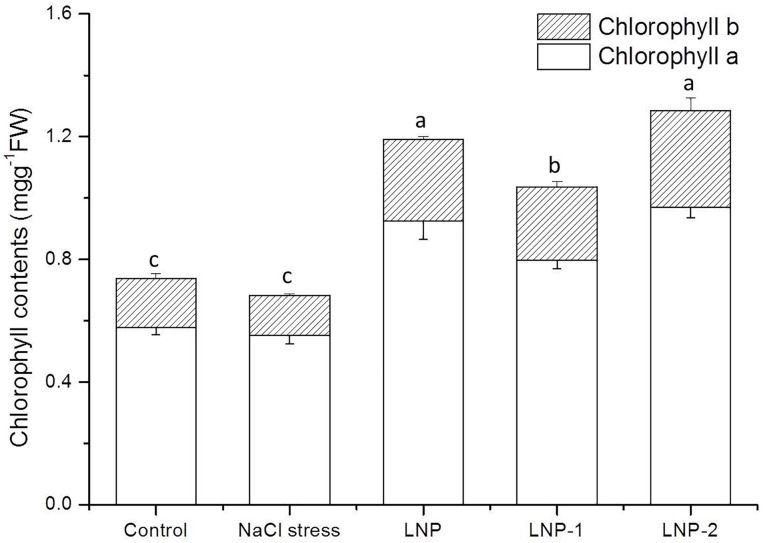
Effect of LNP, LNP-1, and LNP-2 on chlorophyll content in wheat seedlings. Values are the mean ± SD of three replicates. Different letters indicate significant differences at *P* < 0.05.

When plants were exposed to stress, chlorophyll contents tended to decrease significantly. Therefore, chlorophyll content is widely used as an index of abiotic stress tolerance in plants. In the present study, Chl-a and Chl-b content decreased under NaCl stress (Figure 4). LNPs alleviated chlorophyll decline in wheat seedlings under salt stress. The Chl-a content of plants treated with LNP, LNP-1, and LNP-2 was significantly greater at 67.9, 44.7, and 75.9%, respectively than Chl-a of salt-stressed plants (Figure 4). Similarly, the Chl-b content of LNP, LNP-1, and LNP-2 was markedly higher (103.7, 81.9, and 141.1%, respectively), than in salt-stressed seedlings. Moreover, crude LNP and LNP-2 increased chlorophyll content much more than LNP-1 compared to NaCl stressed plants.

#### Soluble Sugar and Proline Content

**FIGURE 5:**
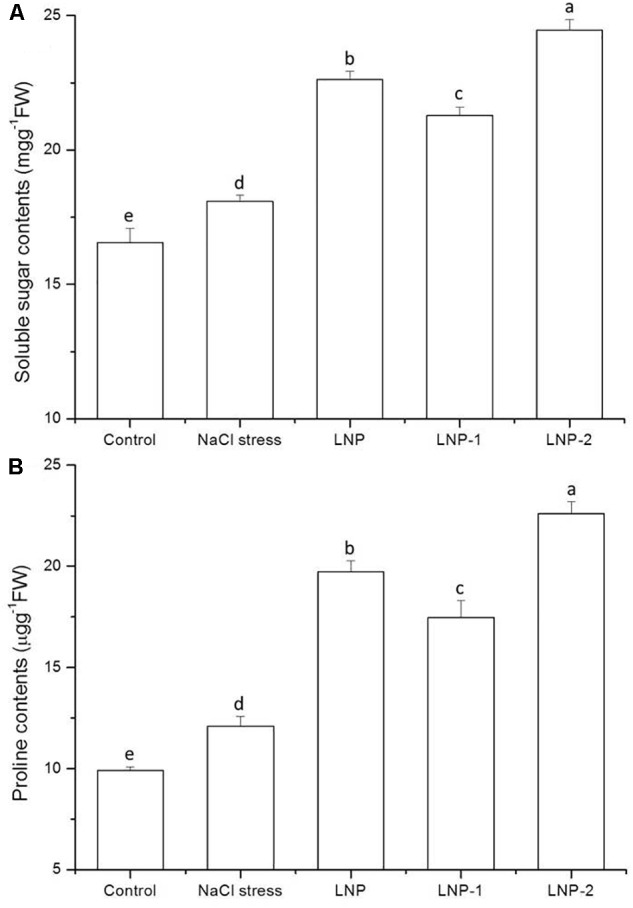
Effect of LNP, LNP-1, and LNP-2 on soluble sugar **(A)** and proline content **(B)** in wheat seedlings. Values are the mean ± SD of three replicates. Different letters indicate significant differences at *P* < 0.05.

In the present study, soluble sugar content increased significantly in wheat seedlings under salt stress compared to the control (Figure 5A). In wheat seedlings treated with LNP, LNP-1, and LNP-2, soluble sugar content increased by 25.2, 17.7, and 35.3%, respectively, compared with salt-stressed plants. Moreover, LNP-2 induced soluble sugar production more strongly than the other treatments. The fact that the soluble sugar content increased in wheat seedlings treated with different LNPs suggests that soluble sugars can help maintain osmotic balance and stabilize cell membranes in plants.

As shown in Figure 5B, in response to NaCl treatment, the proline content in wheat seedling leaves increased significantly by 22.0% relative to the control. There was a significant increase in the proline content of wheat seedlings treated with LNPs. In salt-stressed plants, LNP, LNP-1, and LNP-2 significantly increased the proline content by 63.4, 44.6, and 87.1%, respectively, compared with the NaCl-stressed group. The results showed that LNP-2 promoted proline production and accumulation more strongly than the other treatments.

#### Antioxidant Enzymes Activities

**FIGURE 6:**
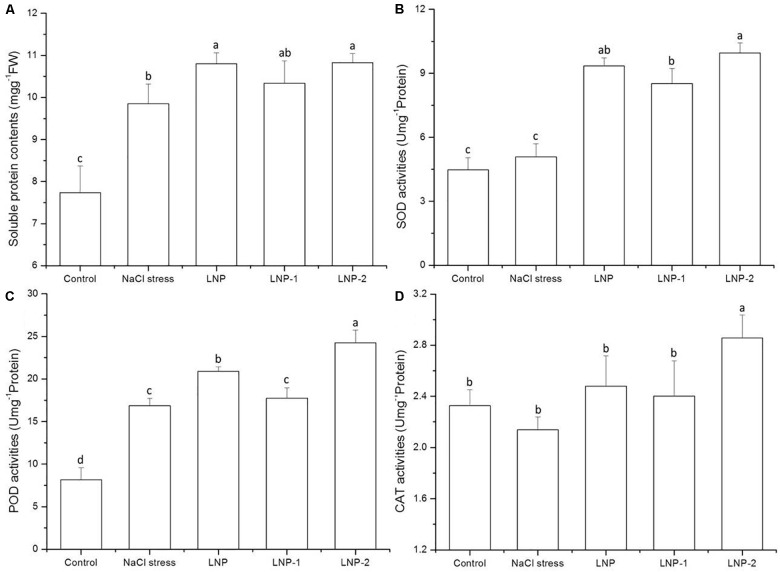
Effect of LNP, LNP-1, and LNP-2 on soluble protein contents **(A)**, SOD **(B)**, POD **(C)**, and CAT **(D)** activities in wheat seedlings. Values are the mean ± SD of three replicates. Different letters indicate significant differences at *P* < 0.05. SOD, superoxide dismutase; POD, peroxidase activity; CAT, catalase.

Soluble protein content significantly increased under salt stress (Figure 6). In wheat seedlings treated with LNP, LNP-1, and LNP-2, the soluble protein content increased by 9.7, 5.0, and 9.9%, respectively, compared to the negative control (Figure 6A). However, no significant difference in soluble protein content was observed between the NaCl-stressed group and the LNP-1 treatment group. In the present study, treatment with the various LNPs increased SOD activity. LNP, LNP-1, and LNP-2 significantly increased the SOD activities by 84.2, 67.9, and 96.0%, respectively, compared to the salt-stressed plants (Figure 6B). Similarly, LNP and LNP-2 increased the POD activities by 24.0 and 43.9%, respectively, relative to the NaCl-stressed plants (Figure 6C). However, there was no significant difference between the NaCl-stressed and the LNP-1-treated group. The CAT activity was only improved by treatment with LNP-2 (Figure 6D). This result suggested that antioxidant enzyme activities in the LNP-2 treatment were significantly higher than those in the other groups.

#### Na^+^ and K^+^ Accumulation in Different Tissues of Wheat Seedlings

**FIGURE 7:**
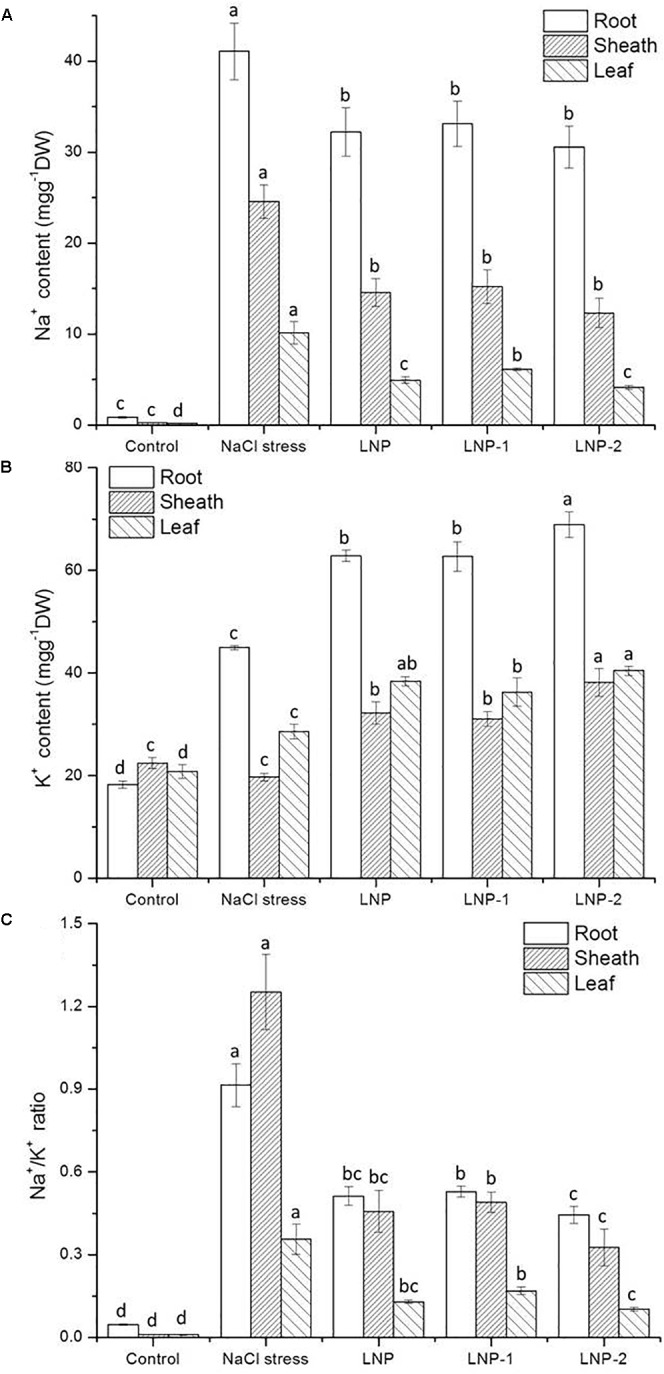
Effect of LNP, LNP-1, and LNP-2 on Na^+^ **(A)**, K^+^ contents **(B)**, and Na^+^/K^+^ ratio **(C)** of root, sheath, and leaf in wheat seedlings. Values are the mean ± SD of three replicates. Different letters indicate significant differences at *P* < 0.05.

The results showed that Na^+^ content in different tissues of wheat seedlings increased significantly under salt stress. In the root, sheath, and leaf, the increase of Na^+^ content in salt-stressed plants was 48.9, 105.6, and 49.7-times of that of the control (Figure 7A), respectively. The K^+^ content in the root and leaf of salt-stressed plants increased by 146.3 and 37.4%, respectively. However, there was a reverse trend in K^+^ content in the sheath of wheat seedlings (Figure 7B). The K^+^ content in the salt-stressed group was slightly lower than that in the control group, but no significant difference was observed. In wheat seedlings treated with LNPs, Na^+^ content was lower than that of salt-stressed plants, but was still higher than that of the control group. The results showed that Na^+^ accumulated in different tissues under salt stress, especially in roots. In wheat seedlings treated with LNPs, Na^+^ content decreased by 21.6, 19.4, and 25.6% in roots; 40.8, 38.2, and 50.0% in sheaths; and 51.3, 39.6, and 59.1% in leaves, respectively. However, there was no significant difference in the Na^+^ content among the LNP groups. In contrast, LNP application increased the K^+^ content in roots, sheaths, and leaves compared with salt-stressed plants. Consequently, a lower Na^+^/K^+^ ratio was observed in the roots, sheaths, and leaves of LNP-treated plants (Figure 7C).

#### Expression of Genes Encoding the Na^+^/K^+^ Transporter

**FIGURE 8:**
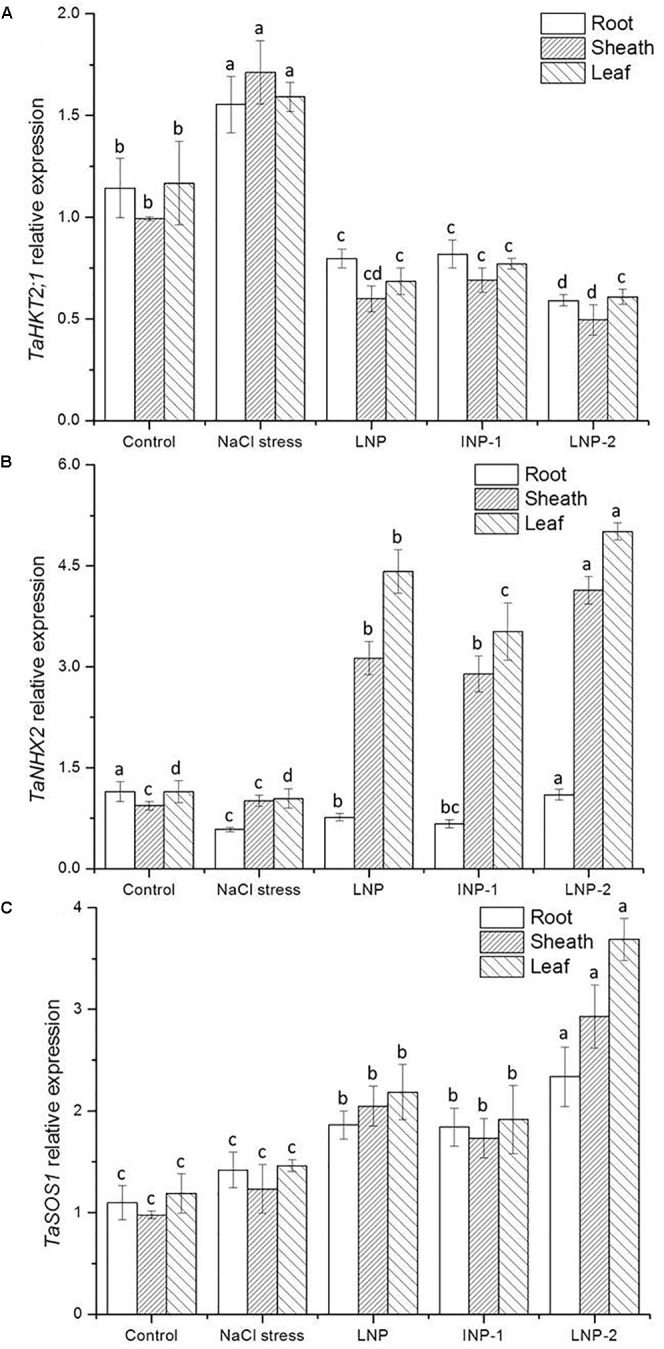
Effect of LNP, LNP-1, and LNP-2 on *TaHKT2;1* **(A)**, *TaNHX2* **(B)**, and *TaSOS1* **(C)** expression of root, sheath, and leaf in wheat seedlings. Values are the mean ± SD of three replicates. Different letters indicate significant differences at *P* < 0.05.

Compared to the control, salt stress induced higher transcript levels of *TaHKT2;1*. Treatment with LNPs significantly down-regulated the expression of *TaHKT2;1* in the roots, sheaths, and leaves (Figure 8A). In contrast, the *TaNHX2* expression was obviously down-regulated in roots of the salt-stressed plants, but there was no significant difference in sheaths and leaves (Figure 8B). After LNP application, the *TaNHX2* expression was down-regulated in roots, but up-regulated in sheaths and leaves. Gene expression was 3.1-, 2.9-, and 4.1-fold in sheaths and 4.2-, 3.4-, and 4.8-fold in leaves in the LNP-, LNP-1-, and LNP-2-treated plants, respectively, compared to the salt-stressed plants. In plants treated with LNP, LNP-1, and LNP-2, the expression of *TaSOS1* was up-regulated by 31.1, 29.6, and 64.6% in roots; 66.4, 40.8, and 138.2% in sheaths; and 49.7, 31.4, and 152.7% in leaves, respectively. Moreover, the transcript levels of *TaSOS1* in plants treated with LNP-2 were significantly higher than those in the other groups (Figure 8C).

## Discussion

Salt stress restricts the growth and development of plants by influencing physiological and biochemical processes, such as osmotic pressure, superoxide ion homeostasis, and antioxidant responses. The seaweed extracts are used in agriculture due to their ability to regulate plant resistance responses to different environmental stresses. Our study demonstrated that exogenous supply of polysaccharides from *L. nigrescens* could alleviate the adverse effects of salt stress on the growth of wheat seedlings through improving antioxidant activities and regulating the efflux and compartmentation of intracellular ions.

The observed significant reductions in fresh and DW indicated that salt stress inhibited growth and caused damage to wheat seedlings. Salinity reduced root length, fresh and DW in agreement with earlier studies (Annunziata et al., 2017). Reduction in root extension rates might come from the marked lowering of root turgor and water potential (Rodriguez et al., 1997). The application of the LNPs could improve the wheat seedling growth parameters (Table 2). However, its mechanism is highly complex and needs to be further discussed.

Malondialdehyde, a marker of lipid peroxidation and cell damage, was significantly increased under salt stress treatments, but low levels of MDA were found in LNPs treated plants (Figure 3A). The results of the present study are similar to the previous findings that exogenous polysaccharides from *Pyropia yezoensis* could scavenge free radicals and prevent salt-stress-related lipid peroxidation (Bose et al., 2014).

Abiotic stresses seriously affect photosynthesis in plants, such as reduction in chlorophyll content, disintegration of chloroplast membranes, and disruption of photosystem biochemical reactions. The present study demonstrated that NaCl stress significantly decreased chlorophyll content in wheat seedlings (Figure 4). Degradation of chlorophyll under abiotic stress is usually related to the accumulation of ROS, which leads to lipid peroxidation of chloroplast membranes. In the current study, salt stress increased the MDA content of wheat seedlings, indicating that membrane lipids were damaged by ROS generation caused by salt stress. However, the MDA levels in LNPs-treated wheat seedling leaves declined compared to those of the NaCl-stressed group. In the present study, improvement in the growth of plants exposed to NaCl stress was determined by the increases in chlorophyll content. It was reported that the seaweed extract enhanced plant chlorophyll content by inducing its synthesis (Khan et al., 2009). The LNPs significantly increased chlorophyll levels in plants under NaCl stress. The results indicated that LNP treatment reduced lipid peroxidation and mitigated the salt-induced decline in chlorophyll content.

Proteins and other macromolecules rapidly decompose due to abiotic stresses. In the process, plant cell membranes are damaged and imbalances in osmotic pressure occur. Sugars act as an osmotic regulator and reduce membrane permeability, specifically by reducing water potential in cells and stabilizing cell membranes. Under abiotic stress, the contents of compatible osmolytes such as proline, betaine, and free amino acids increased in the cytoplasm to prevent cytoplasm dehydration caused by salt stress. Proline not only acts as an osmotic protector under abiotic stress but also regulates osmotic potential, stabilizes cell structure, and reduces damage to the photosynthetic apparatus. Zhang et al. (2017) pinpoint that the proline metabolic pathway showed significant differences in response to salt stress. Proline induces the expression of salt stress-responsive genes, which promotes the adaptation of plants to salt stress. Proline may have a protective effect as it can induce antioxidant enzymes to scavenge ROS. Indeed, application of exogenous proline improved antioxidant activities, reduced the oxidative damage, and lipid peroxidation levels (Hasanuzzaman et al., 2014).

In this study, high proline content indicated that salt stress-induced proline biosynthesis in plants helped to ameliorate the osmotic imbalance. LNPs further increased proline content in plants under salt stress (Figure 5B). The proline content of the LNP-2 treatment plants was considerably higher than that of the other groups, indicating stronger osmotic regulation ability in the LNP-2-treated plants.

Salt stress leads to crop yield loss because of imbalances in mineral nutrient concentrations and osmotic effects, triggering excess ROS production (Mittler et al., 2004). Under normal physiological conditions, ROS are constantly produced by aerobic metabolism in chloroplasts, mitochondria, and peroxisomes. However, ROS overproduction as a consequence of stress exposure can lead to oxidative damage to lipids, proteins, nucleic acids, and cause membrane dysfunction and cell death (Mittler et al., 2004).

It has been reported that regulation of ROS generation in plants may effectively protect against oxidative damage and increase stress tolerance (Bose et al., 2014). An antioxidant system consisting of a variety of enzymes and small molecules protects plants from oxidative damage. Antioxidant enzymatic activities and accumulation of antioxidants play an important role in the inhibition of membrane protein and lipid peroxidation. Many studies have shown that SOD, POD, and CAT are closely related to salt resistance in plants (Qiu et al., 2014). SOD detoxifies O_2_ ∙radicals by forming H_2_O_2_, which is also toxic and must be eliminated by the concerted actions of CAT and POD.

In wheat, several studies showed that wheat plants alter the activity of antioxidant enzymes such as SOD, CAT, APX, POX, and GR under different abiotic stresses attempt to defend against oxidative damage (Caverzan et al., 2016). Most antioxidant enzymes increased their activity in response to the major abiotic stresses faced by wheat plants. For example, in wheat, an increase in the SOD transcript was observed under different heat shock treatment (Kumar et al., 2013). This is consistent with the results of the present study, since SOD and POD activities were significantly higher in salt-stressed plants than in the control. Plants treated with LNPs under NaCl stress also showed relative increases in SOD, POD, and CAT activities. The activation of these enzymes leads to wheat seedlings protection against oxidative damage. These results indicate that LNPs effectively induce ROS scavenging in wheat seedlings by modulating their antioxidant enzyme activities. Therefore, LNPs may enhance defense responses in plants under salt stress. In general, LNP-2 was significantly more effective at inducing antioxidant activity and ROS scavenging than the other groups.

In the present study, increased proline content and decreased MDA in plants after LNP pre-treatment was consistent with the increase of the antioxidant enzyme indicating that the pre-treatment with LNP can induce proline, regulates the enzymes, and decreases lipid peroxidation, thus reducing ROS directly and protects plants from salt stress.

The Na^+^ content in all wheat tissues of control group was negligible, and treatment with 150 mM NaCl for 10 days resulted in a large amount of Na^+^ accumulated in roots. Under salt stress, excess Na^+^ can lead to an imbalance in cellular Na^+^ and K^+^ homeostasis, which play an important role in the growth and development of higher plants (Zhang et al., 2018). Sodium within the plant has a devastating effect on the metabolism of cytoplasm and organelles, because it tends to replace potassium in key enzymatic reactions. Restricting the transport and accumulation of Na^+^ in leaves is the most important adaptation of plant to salt stress (Mekawy et al., 2015). Wheat is a classical “salt excluder,” characterized by low rates of Na^+^ transport to the shoot, thus keeping mesophyll cells as Na^+^-free as possible (Munns and James, 2003; Colmer et al., 2005; James et al., 2006). In this study, the Na^+^ content was significantly lower than that in roots, indicating that plants restricted the transport of Na^+^ from roots to leaves. Moreover, the Na^+^ content of leaves treated with exogenous LNPs was significantly lower compared to that of salt-stressed plants. Wheat seedlings treated with LNPs selectively excluded Na^+^ from leaves, which are the basis tissue of photosynthesis (Lekshmy et al., 2015). Wheat seedlings treated with LNP and LNP-2 exhibited lower leaf Na^+^ content and thus should be better adapted to salt stress.

However, Na^+^ exclusion is not always sufficient to improve plant salt tolerance. In addition to low Na^+^ transport rates to the shoots, a high selectivity for K^+^ also plays an important role in salt tolerance in wheat. K^+^ is one of the most abundant macronutrients in plants and is essential for maintaining membrane potential and swelling pressure, activating enzymes, regulating osmotic pressure, stomatal movement, and orientation (Laurie et al., 2002). Maintenance of a high cytosolic K^+^:Na^+^ ratio is a key feature of plant salt tolerance (Cuin et al., 2008). Under salt stress conditions, plants are affected by K^+^ accumulation due to the competitive inhibition of Na^+^ uptake, which often leads to a high Na^+^/K^+^ ratio that disrupts the intracellular balance (Mekawy et al., 2015). In this study, the cytosolic Na^+^:K^+^ ratio rose dramatically under salt stress due to excessive Na^+^ accumulation in the cytosol. However, K^+^ accumulation in LNP-treated plants was accompanied by a lower Na^+^/K^+^ ratio, thus increased salt tolerance. Moreover, the Na^+^/K^+^ ratio in LNP-2-treated plants was lower than that of other groups but without obvious difference with the LNP group. The transmembrane transport of Na^+^ and K^+^ in plants is mediated by several types of transporters and/or channels (Yao et al., 2010), some of which are closely related to Na^+^ exclusion in leaves, including high-affinity K^+^ transporters (HKTs). In bread wheat, *TaHKT2;1* has been confirmed and is assumed to function in Na^+^ uptake from the soil (Ariyarathna et al., 2014). Kumar et al. (2017) previously studied the expression of *TaHKT2;1* in contrasting wheat genotypes. The results showed that the transcript levels of *TaHKT2;1* were up-regulated in the shoots of the salt-sensitive genotype, but down-regulated in the salt-tolerant genotype. Furthermore, under salt stress an increase in cytosine methylation down-regulated *TaHKT 2;1* and *TaHKT2;3* expression in salt-tolerant wheat, thereby improving salt tolerance. This indicated that its inhibition of gene expression was associated with salt tolerance. In the present study, salt stress up-regulated the expression of the *TaHKT2;1* gene, while LNPs significantly down-regulated the expression of *TaHKT2;1* in roots. These results suggest that *TaHKT2;1* inhibits the absorption of Na^+^ from the soil and thus decreases Na^+^ content and Na^+^/K^+^ ratio in leaves.

In addition to limiting Na^+^ entry into cells, salt tolerance mechanisms of plants also include Na^+^ exclusion and compartmentalization of Na^+^ into vacuoles to prevent Na^+^ accumulation (Yamaguchi et al., 2013). The Salt Overly Sensitive 1 (*SOS1*) antiporter is a plasma membrane Na^+^/K^+^ antiporter that mediates the efflux of cytosolic Na^+^ in roots and regulates the transportation of Na^+^ from root to shoot to maintain appropriate K^+^/Na^+^ ratio in leaves (Qiu et al., 2002). NHX, an Na^+^/K^+^ antiporter localized in vacuolar membranes, can mediate Na^+^ regionalization into vacuoles (Gaxiola et al., 2001). Under 200 mM NaCl concentration, *SOS1* and *NHX1* expression in salt-tolerant wheat genotypes increased significantly, thereby improving Na^+^ exclusion and lowering the Na^+^/K^+^ ratio (Lekshmy et al., 2015). In this study, *TaSOS1* and *TaNHX2* overexpression were significantly up-regulated in wheat seedlings treated with LNPs. These seedlings showed a considerably higher resistance to high NaCl concentrations. In general, *TaSOS1* and *TaNHX2* transcripts in plants treated with LNP-2 were significantly higher than those of the other groups. These results indicated that LNP-2 effectively supported the efflux and regionalization of Na^+^, thus alleviating salt stress damage.

Although there have been many reports that algae extracts contain biological stimulants and protective agents, due to the diversity and complexity of these extracts, it is difficult to determine the active compounds. The effects of biostimulants have generally been attributed to the presence of phytohormones, organic molecules, phenolic compounds, amino acids, and bioactive secondary metabolites (Stasio et al., 2018). Apart from these compounds, polysaccharides may also contribute to the observed beneficial effects. The present study proved that polysaccharides derived from *L. nigrescens* and its purified fractions can improve the resistance of plants to salt stress.

We found that polysaccharide activity mainly depends on MW and sulfate content. This conclusion is in line with the results of previous studies showing that both properties determine the biological activities of fucoidan from the brown seaweed *Adenocystis utricularis* (Ponce et al., 2003). However, Zha et al. (2016) stated that a low-MW polysaccharide isolated from *Laminaria japonica* has effective scavenging activities on ROS *in vitro*. This is in agreement with the results of Qi et al. (2006), who demonstrated that the low-MW polysaccharide fraction prepared from *Ulva pertusa* had stronger reducing power due to the higher number of reducing and non-reducing ends. Sangha et al. (2010) stated that the efficiency of inductive activity depended on the species and its sulfate degree of carrageenan. The pre-treatment of λ-carrageenan with high sulfation induced resistance to *S. sclerotiorum* resulting in less foliar damage while the ι-carrageenan with low sulfation increased the severity of the disease. In the current study, LNP-2 (40.2 kDa) treatment was more effective at inducing salt resistance than the LNP (45.4 kDa) and LNP-1 (63.9 kDa) treatments. This result may suggest a relationship between salt resistance and polysaccharide MW and the amount of sulfates. Specifically, salt resistance capacity seemed to be higher with lower MW and higher sulfate content. The presence of LNP-2 in LNP may explain the higher salt-resistance in the LNP treatment than in the LNP-1 treatment.

## Conclusion

In the present study, polysaccharides extracted from *L. nigrescens* (LNP) have similar characteristics to species of the genus *Lessonia*. Crude polysaccharides were further separated and fractionated and two acidic polysaccharides (LNP-1 and LNP-2) from crude polysaccharide LNP were obtained. Moreover, the salt-resistance activity of plants induced by the three polysaccharides was studied. The results showed that all of the three polysaccharides could induce plant’s resistance to salt stress, and LNP-2 showed more effective plant salt-resistance activity than the other groups. It can be concluded from this work that both the MW and the sulfate degree contribute to the salt-resistance activity of polysaccharides from *L. nigrescens*. The results of this study can make a positive contribution to the cultivation and promotion of crop and utilization of algae resources.

## Author Contributions

PZ conceived the study, did most of the experimental work, and wrote the manuscript. LM and HZ preparation of polysaccharide. YY chemical analysis of polysaccharide. XL plant growth analyses and study the effect of polysaccharides on wheat seedlings under salt stress. CZ and YL reviewed and edited the manuscript.

## Conflict of Interest Statement

The authors declare that the research was conducted in the absence of any commercial or financial relationships that could be construed as a potential conflict of interest.

## Supplementary Material

FIGURE S1 The MW of LNP, LNP-1, and LNP-2 measured by HPLC with dRI detector.

FIGURE S2 Effects of LNP, LNP-1, and LNP-2 on growth of wheat seedlings.
