# Supplementary figures and images for: Polysaccharides Derived From the Brown Algae Lessonia nigrescens Enhance Salt Stress Tolerance to Wheat Seedlings by Enhancing the Antioxidant System and Modulating Intracellular Ion Concentration
Source: Front Plant Sci. 2019 Jan 31;10:48. doi: 10.3389/fpls.2019.00048 (PMC6365471; doi:10.3389/fpls.2019.00048)

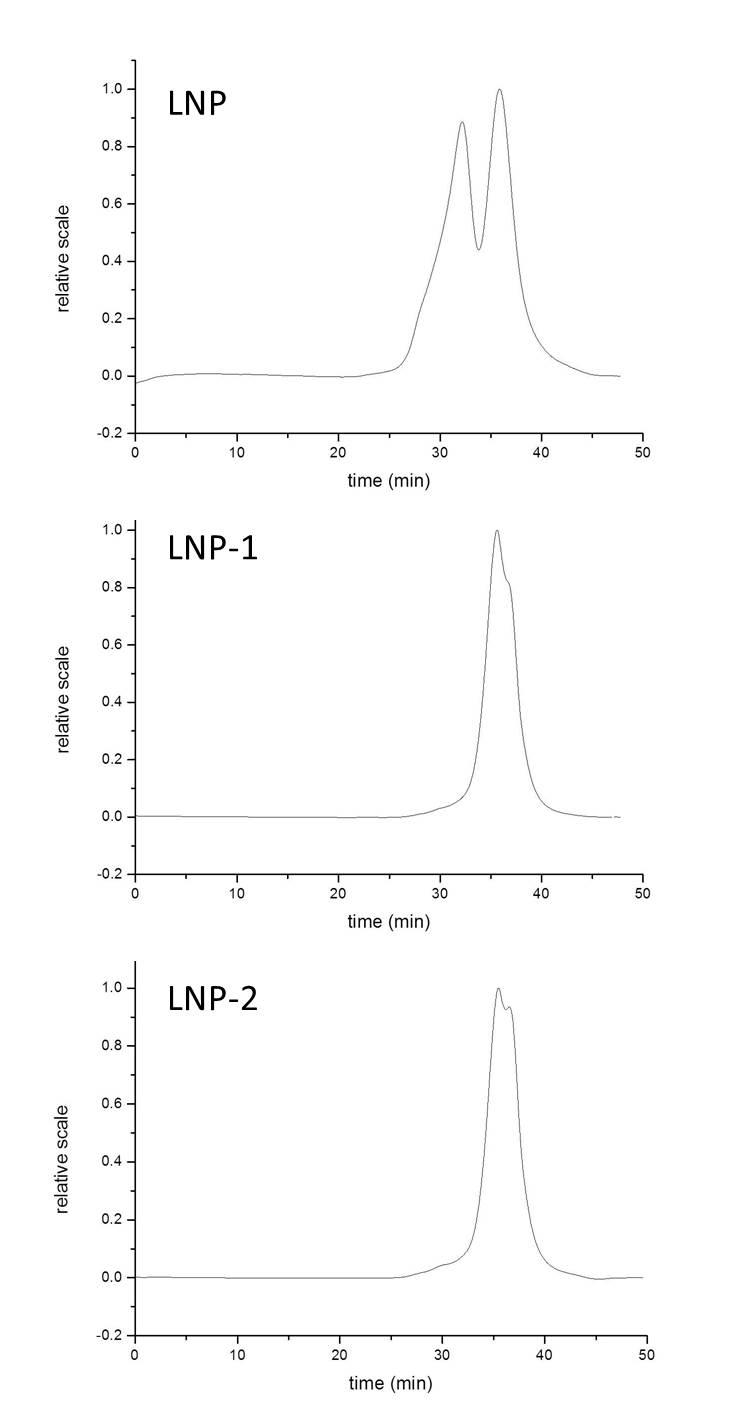

Supplement: FIGURE S1 — The MW of LNP, LNP-1, and LNP-2 measured by HPLC with dRI detector. [file Image_1.JPEG]

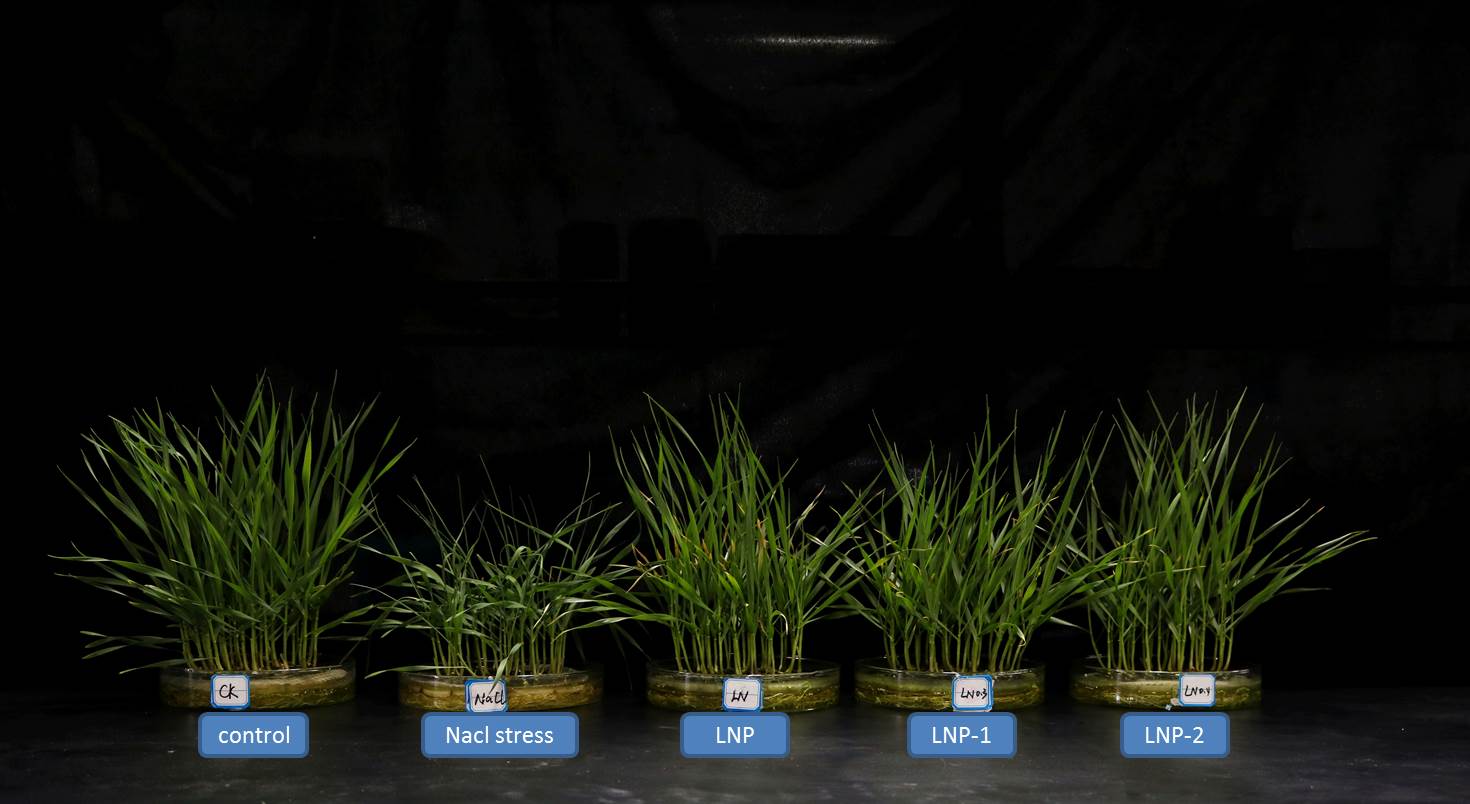

Supplement: FIGURE S2 — Effects of LNP, LNP-1, and LNP-2 on growth of wheat seedlings. [file Image_2.JPEG]
