# Supplementary material for: Polysaccharides Derived From the Brown Algae Lessonia nigrescens Enhance Salt Stress Tolerance to Wheat Seedlings by Enhancing the Antioxidant System and Modulating Intracellular Ion Concentration
Source: Front Plant Sci. 2019 Jan 31;10:48. doi: 10.3389/fpls.2019.00048 (PMC6365471; doi:10.3389/fpls.2019.00048)
Supplement: Supplementary file 3 [file Table_1.DOCX]

Table S 1 Primers used for quantitative real-time RT-PCR.

| Gene | Accession | Primer pairs |
| --- | --- | --- |
| *TaNHX2* | AY040246 | F：TTCCAACCAGAACCAACCC  R：GTCCTTCATCGCTGAGACTTTT |
| *TaSOS1* | AY326952 | F：CGGAGGGTGGATTGAACGA  R：GCAGGGCGGTAGGAGAAGAT |
| *TaHKT2;1* | KR422358.1 | F：TATGTGATGAGTCGCAGCTTGAA  R：GCAACAAGAGGCCTGAATTCTTT |
| β-actin | AB181991 | F：CTCTGACAATTTCCCGCTCA  R：ACACGCTTCCTCATGCTATCC |
